# Revealing the mechanism of quinoa on type 2 diabetes based on intestinal flora and taste pathways

**DOI:** 10.1002/fsn3.3710

**Published:** 2023-09-23

**Authors:** Chun‐Yan Zheng, Tian An, Zheng‐Ting Liang, Bo‐Han Lv, Yu‐Tong Liu, Xue‐Hong Hu, Yue‐Lin Zhang, Nan‐Nan Liu, Si‐Yu Tao, Ru‐Xue Deng, Jia‐Xian Liu, Guang‐Jian Jiang

**Affiliations:** ^1^ Traditional Chinese Medicine School Beijing University of Chinese Medicine Beijing China; ^2^ School of Traditional Chinese Medicine Capital Medical University Beijing China; ^3^ Traditional Chinese Medicine School Xinjiang Medical University Xinjiang China; ^4^ Gansu Pure High‐Land Agricultural Science and Technology Limited Company Lanzhou China; ^5^ Zhong Li Science and Technology Limited Company Beijing China

**Keywords:** diabetes, intestinal flora, molecular docking, network pharmacology, quinoa, taste receptor

## Abstract

To investigate the antidiabetic effects and mechanisms of quinoa on type 2 diabetes mellitus (T2DM) mice model. In this context, we induced the T2DM mice model with a high‐fat diet (HFD) combined with streptozotocin (STZ), followed by treatment with a quinoa diet. To explore the impact of quinoa on the intestinal flora, we predicted and validated its potential mechanism of hypoglycemic effect through network pharmacology, molecular docking, western blot, and immunohistochemistry (IHC). We found that quinoa could significantly improve abnormal glucolipid metabolism in T2DM mice. Further analysis showed that quinoa contributed to the improvement of gut microbiota composition positively. Moreover, it could downregulate the expression of TAS1R3 and TRPM5 in the colon. A total of 72 active components were identified by network pharmacology. Among them, TAS1R3 and TRPM5 were successfully docked with the core components of quinoa. These findings confirm that quinoa may exert hypoglycemic effects through gut microbiota and the TAS1R3/TRPM5 taste signaling pathway.

## Graphical abstract

The results of this study showed that quinoa has a positive effect on the recovery of intestinal microbiota disorder in type 2 diabetes mice.

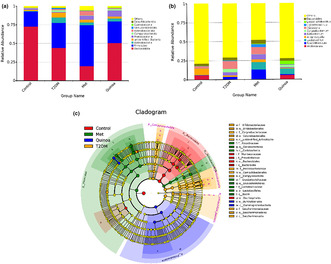

## 1 INTRODUCTION

Diabetes mellitus is a complicated endocrine disease characterized by hyperglycemia (Shao & Espenshade, 2012), which is an attack on human health following cancer and cardiovascular disease (Singh, 2016). Many factors, including genetic, obesity, and intestinal microbiota, are shown closely related to the progression of T2DM (Jung et al., 2016; Ling & Rönn, 2019; Scherer Philipp & Hill Joseph, 2016; Yang et al., 2017; Zhang et al., 2013). It is estimated that T2DM will afflict the health of nearly 592 million people by 2035 (Guariguata et al., 2014). Oral hypoglycemic drugs (such as metformin, sulfonylureas, α‐glucosidase inhibitors, and glinides) and insulin therapy are the main clinical treatments that are used to regulate blood sugar and relieve the symptoms of T2DM (Aschner, 2020; Dowarah & Singh, 2020; Lv et al., 2020; Sanchez‐Rangel & Inzucchi, 2017). However, it is associated with many negative influences, such as hypoglycemia, gastrointestinal tract reaction, and cardiovascular risk (Lv et al., 2020; Scholze, 2017). Recently, studies have shown that regulating dietary structure plays an important role in hyperglycemia (Forouhi et al., 2018; Ojo, 2019).

Quinoa (Chenopodium quinoa willd) is a historical pseudocereal that originated from the Cordillera‐Andes Mountains of South America (Valenzuela Zamudio & Segura Campos, 2022). As “one of the grains of the twenty‐first century” (Vilcacundo & Hernández‐Ledesma, 2017), quinoa has a high nutritional value. It contains a variety of functional components, such as total phenols, saponins, flavonoids, and polysaccharides, which can improve human nutrition level and prevent a variety of diseases. Dietary fibers also have been considered as one of the major bioactive ingredients in quinoa (Chen, Xiong, et al., 2021; Hu et al., 2023; Liu et al., 2020; Liu, Wang, et al., 2022). Studies have also shown that quinoa can be used to treat obesity, metabolic syndrome, and T2DM (Lin et al., 2019; van den Driessche et al., 2018; Wang, Tao, et al., 2022). The compositions contained in quinoa, such as polyphenols, bioactive peptides, and phytoecdysteroids, have excellent hypoglycemic effects (Graf et al., 2014; Valenzuela Zamudio & Segura Campos, 2022; Zhang et al., 2022).

Network pharmacology is an advanced method based on multi‐disciplinary theory that uses computer, high‐throughput data screening, and other technologies to predict the relationship between drugs and diseases (Hopkins, 2008; Ye et al., 2016). At present, it has been used in the prediction of cardiovascular diseases, pulmonary diseases, and metabolic diseases harmoniously (Huang et al., 2021; Li et al., 2021; Wang, Zhu, et al., 2022). To predict the mechanism of quinoa prevention and treatment of diabetes more scientifically, this research, through the correlation analysis of network pharmacology and molecular docking, discovers the potential pathway of quinoa to play a role from a multidimensional perspective, providing useful clues for further research.

Nutritional sensing refers to the process by which cells perceive and transmit nutrients, such as sugars, amino acids, lipids, and other simple organisms (Efeyan et al., 2015). It has been suggested that nutrient receptors for monosaccharides, such as GLUcose (TAS1R2/TAS1R3 heterodimer) and amino acids (TAS1R1/TAS1R3 heterodimer), are not only present in oral taste buds but are also expressed in the intestinal tract (Rasoamanana et al., 2012). The combination of these gastrointestinal nutrients with taste receptors leads to a series of signal transduction, which regulates the metabolic balance of the organism. Studies have shown that taste receptor TAS1R1/TAS1R3 can mediate the signal transduction induced by aliphatic amino acids (Tolhurst et al., 2012), and the imbalance of branched‐chain amino acids (BCAAs), as a pathological marker, may lead to the occurrence of obesity and T2DM and other endocrine diseases (Giesbertz & Daniel, 2016; Le Couteur et al., 2020; Lynch & Adams, 2014). The heterodimer formed by Tas1R3 binding to Tas1R1 has been shown to regulate the amino acid‐induced insulin secretion in pancreatic β‐cells (Oya et al., 2011). TRPM5 plays a major role in glucose‐induced insulin secretion beyond membrane depolarization, and its dysfunction may lead to the occurrence and development of type 2 diabetes (Brixel et al., 2010). Research has shown that the taste receptor subunit TAS1R3 and its downstream molecule TRPM5 can further regulate the secretion of GLP‐1 after sugar intake, leading to impaired glucose regulation, dysfunction of pancreatic islet cells, and insufficient insulin secretion (Zhang et al., 2016).

Related studies have reported that under the intervention of quinoa, beneficial bacteria could get a promotion, but the potential mechanism remains unclear (Navarro del Hierro et al., 2019). In addition, the precise link between intestinal microbiota, nutrient sensing, and taste pathway is still obscure. Herein, we use T2DM C57BL/6 mouse model to analyze the biological indicators, intestinal flora, and related gene expression which are supplied by quinoa; we also learn from network pharmacology and molecular docking to explore and verify the multi‐target and multi‐pathway regulation of quinoa on diabetes to provide a scientific basis for its development and utilization of hypoglycemic diet.

## 2 MATERIALS AND METHODS

### 2.1 Diets and animals

The ordinary diet, high‐fat diet (HFD, conventional feed: 66.5%; lard: 10%; carbohydrates: 20%; bile salt: 0.2%; cholesterol: 2.5%) and quinoa were all provided by Beijing HFK Bioscience Co., LTD. Healthy 4–5‐week‐old C57BL/6 male mice in this study were purchased from Beijing Vital River Laboratory Animal Technology Limited Company, and all the animals were reared at a standard animal feeding room (temperature: 23 ± 1°C; 40% ± 10% humidity, 12 h/12 h light/dark cycle) in Beijing University of Chinese Medicine (BUCM). All experiments were approved by the animal ethics committee of BUCM. The experiments applied in this study were performed according to animal protection guidelines.

### 2.2 T2DM induction and intervention procedure

Twenty‐four 4–5‐week‐old male mice were given general meals and diet balance for adaptive feeding for 1 week. And six mice were randomly divided into a normal control group to receive an ordinary diet. The other 18 mice were fed HFD for 4 weeks and then fasted for 12 h before intraperitoneal injection of STZ (110–120 mg/kg) to induce the T2DM model. After 3 days, the T2DM mouse model was identified as successful if fasting glucose level was ≥11.1 mmoL/L and was randomly divided into the following three groups: positive control group (the metformin group), quinoa group, and T2DM model group. The metformin group was fed orally gavage once a day (100 mg/kg body weight/day), the quinoa group was fed quinoa (2 g/day) for 16 weeks, and all mice were fed the previous diet during this time. Fasting blood glucose (FBG) levels were recorded at the end of each week. Pancreas and colon tissues were selected, and part of them were fixed with 10% formalin for histological examination. The remainder of colon tissue was immediately stored at −80°C for the following analysis.

### 2.3 Biochemical indexes analysis

At the end of the experiment, the eyeball of mice was picked to draw blood for further detection. The blood was stored in evacuated tubes for centrifugation after 2 h stewing at room temperature to take the upper serum supernatant. The levels of triglyceride (TG), glucose (GLU), fasting insulin (FINS), low‐density lipoprotein cholesterol (LDL‐C), high‐density lipoprotein cholesterol (HDL‐C), and total cholesterol (TC) levels were determined using an automatic biochemical analyzer.

### 2.4 Hematoxylin and eosin staining

The pancreas and colon tissues of mice were selected and fixed in 10% formalin for 24 h, dehydrated in a gradient manner, and embedded in paraffin for sectioning, stained with hematoxylin and eosin, and images were captured to evaluate the histopathological changes of pancreas and colon.

### 2.5 IHC and Western blot analysis

The paraffin‐embedded colon was sliced at 4–5 μm with a rotary microtome and placed in an oven at 37°C for 12 h, and then degreased with xylene and hydrated with gradient ethanol. Sequentially, slides were incubated with antigen retrieval solution for 8 min, and we then added rabbit anti‐TRPM5 (1:600), rabbit anti‐TAS1R3 (1:600), and rabbit anti‐GLP‐1 (1:500) at 4°C overnight. Afterward, the slides used for IHC staining were incubated with the secondary antibody for 30 min, followed by DAB and hematoxylin staining. The tissues were examined and photographed by laboratory microscopy. Immunohistochemical positive reaction intensity was analyzed by image‐J6.0 software and expressed as an AOD value. Western blotting was used to detect the expression of TRPM5, TAS1R3, and GLP‐1 proteins in the colon. The colonic protein supernatant was extracted using a tissue protein extraction kit (Servicebio Biotechnology) and the protein concentration was determined using a BCA protein assay kit (Thermo Fisher Scientific). Proteins were separated by 10% SDS‐PAGE gel and then transferred to the PVDF membrane. Then, the latter was blocked with 5% (w/v) skim milk for 1.5 h, incubated with rabbit anti‐TRPM5 (1:1000), rabbit anti‐TAS1R3 (1:1000), and rabbit anti‐GLP‐1 (1:1000) at 4°C overnight. The membrane was gently incubated together with the appropriate secondary antibodies and the bands were visualized with an enhanced chemiluminescence (ECL) reagent. The strip density was quantified by Image J software, and the corresponding β‐actin was used as the internal control for normalization.

### 2.6 Effects of quinoa on the dynamic profile of intestinal microflora

The mice in the four groups were placed in plastic cages separately, and the cages were covered with gauze and the mice were allowed to defecate naturally. The feces of the mice in the four groups at 00 weeks and 16 weeks were taken, and then the feces were quickly loaded into the frozen storage tube with sterilized tweezers. All operating procedures were performed with strict adherence to the principle of sterility. After that, the freeze‐storage tube containing mouse feces was transferred to a −80°C cryogenic refrigerator with a dry ice box for reserve, and then transported to Novogene Co. LTD for preservation and subsequent analysis. 16S rRNA gene sequencing was performed on Illumina MiSeq and data obtained from Illumina MiSeq amplicon sequencing were analyzed using QIIME 2. Sequences with ≥97% similarity were assigned to the same operational taxonomic unit (OTU) by the UCLUST algorithm in QIIME. Tax4Fun was used to generate predicted KEGG annotations. Spearman correlation was used to analyze bacterial community and environmental factors.

### 2.7 Network pharmacology of quinoa on diabetes mellitus

The chemical constituents of quinoa were collected from PubMed and CNKI. The structural formulas of the components were from PubChem (https://pubchem.ncbi.nlm.nih.gov/pccompound/), Chemsrc (WWW. Chemsrc. Com), Organchem (WWW. Organchem. CSDB. Cn). Then, we imported the obtained formula into the SwissADME platform (http://www.swissadme.ch/), and two or more “Yes” ingredients were identified as potential active ingredients in quinoa based on GI absorption (High) and five drug properties (Lipinski, Ghose, Veber, Egan, and Muegge). Then, we import the potential chemical structural formulas of the active ingredients into SwissTargetPrediction (http://www.swisstargetprediction.ch/). Finally, the prediction targets of potential active ingredients were obtained with species as “homo sapiens,” probability >0 as the screening criterion. Ultimately, the potential targets of quinoa were obtained after being standardized by the UniProt database (https://www.uniprot.org/) with the non‐human samples removed. We could obtain therapeutic targets for diabetes from the GeneCard (https://www.genecards.org/), OMIM (https://omim.org/), DisGeNET (http://www.disgenet.org), and DrugBank (https://go.drugbank.com/). Finally, the cross‐network between the quinoa target and diabetes target was analyzed by drawing a Venn diagram (Venny 2.1.0, https://bioinfogp.cnb.csic.es/tools/venny), which represents the potential target of quinoa to ameliorate diabetes. The selected common targets were enriched and imported into the Metascape database for data visualization and KEGG pathway analysis. The “component‐target‐pathway” network diagram of related amino acid metabolism was constructed with Cytoscape3.9.1.

### 2.8 Molecular docking of quinoa on diabetes mellitus

Molecular docking was performed as follows: (1) For ligand molecule preparation, structural information of quinoa components was obtained through PubChem database (https://pubchem.ncbi.nlm.nih.gov/). (2) For receptor molecule preparation, the crystal structure of the proteins was obtained through the RCSB PDB website (http://www.rcsb.org/), and then water molecules and small‐molecule ligands were removed using PyMOL software. AutoDockTools software was used to add nonpolar hydrogens, Gasteiger charges were calculated, and the receptors were prepared and saved in PDBQT format. (3) Molecular docking was performed with the help of PyMOL 2.5.2 software plug‐in to find the docking active site, and the processed components were molecularly docked with the target protein using Autodock Vina 1.2.0 software, and the docking results were visualized using PyMOL 2.5.2 software.

### 2.9 Statistical analysis

The data are expressed as mean ± SD. Statistical analysis was performed for the normality and homogeneity of variance for each group and one‐way analysis of variance (ANOVA) was used to compare differences between groups. When the data conformed to normality and homogeneity of variance, the LSD test was used. When the data conformed to normality but not homogeneity of variance, Dunnett's T3 test was used. When the data were not normally distributed, the nonparametric tests Kruskal–Wallis and Mann–Whitney *U* tests were used. Differences were considered significant if *p* < .05.

## 3 RESULTS

### 3.1 Quinoa reduced hyperglycemia in T2DM mice

#### 3.1.1 Quinoa regulates serum glucose in T2DM mice

**FIGURE 1:**
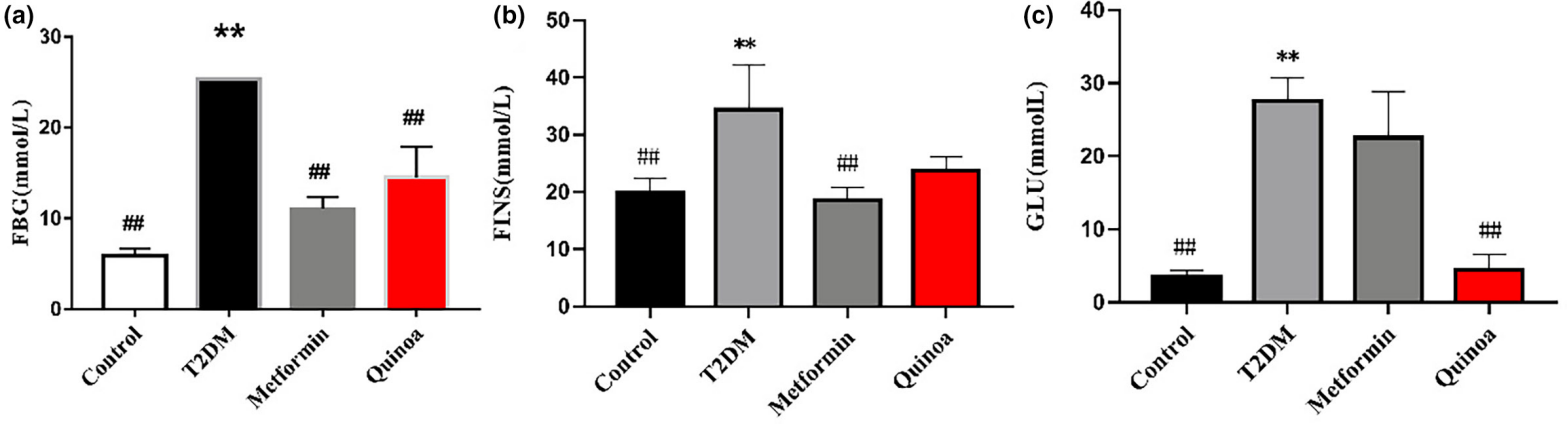
Quinoa regulates serum glucose in T2DM mice. (a) The fasting glucose (FBG) concentrations, (b) The fasting serum insulin (FINS) concentrations, (c) Blood glucose (GLU) concentrations. Values are expressed as mean ± SD (*n* = 6). **p < .01 compared with the control group; ^##^ *p* < 0.01 versus the T2DM group.

Compared with the control group, the levels of FBG, FINS, and GLU were significantly increased in the T2DM group (Figure 1, *p* < .01). After the intervention of metformin and quinoa, the level of FBG presented with a significant decrease compared to that in the T2DM group (Figure 1a, *p* < .01). The FINS level was decreased in the metformin group statistically; while there was a tendency to be lower in the quinoa group compared to the model group, the difference was not statistically significant (Figure 1b). Moreover, the level of GLU was significantly lower in the quinoa group than in the model group (Figure 1c, *p* < .01).

#### 3.1.2 Quinoa ameliorated serum lipids in the T2DM mice

**FIGURE 2:**
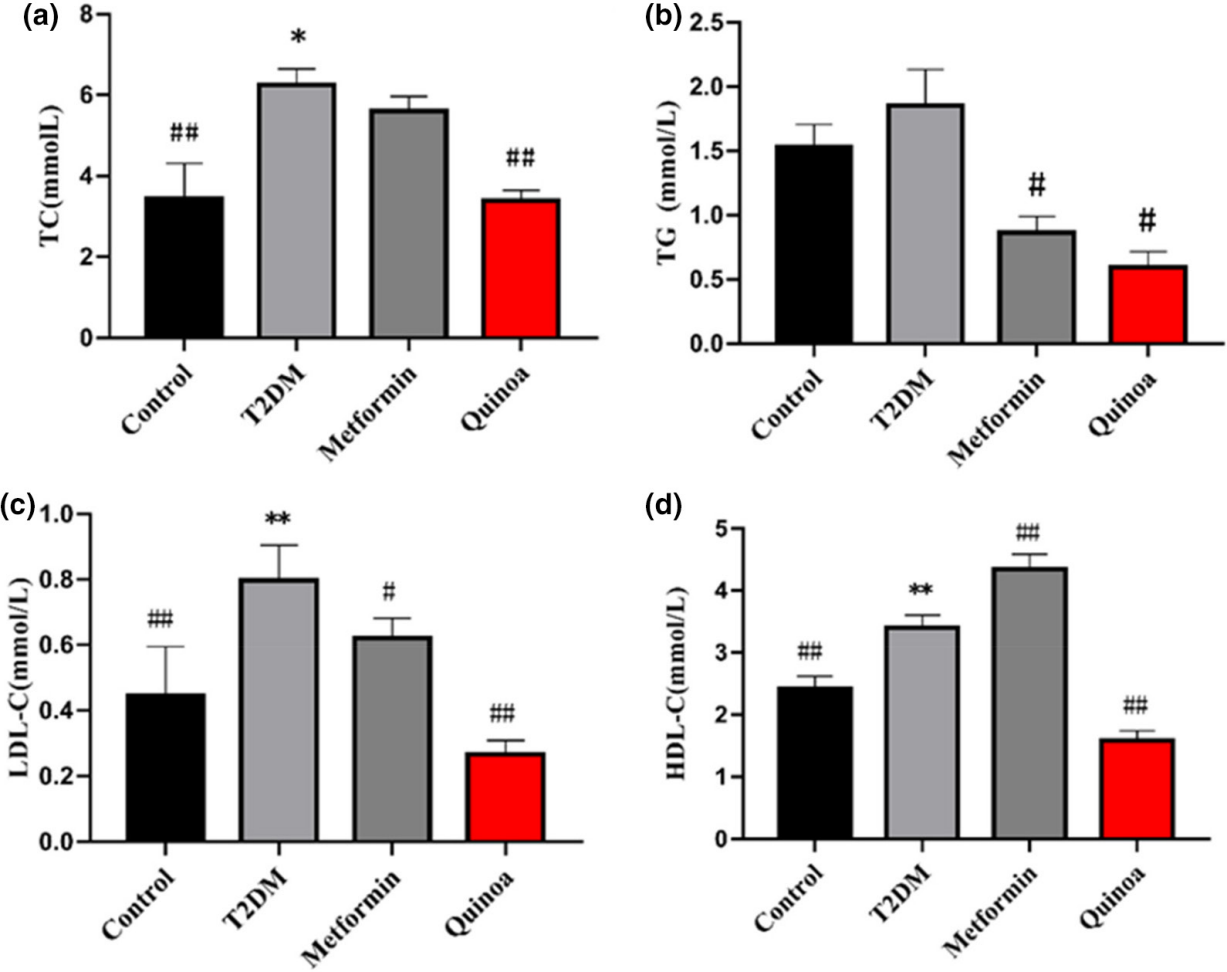
Quinoa ameliorated serum lipids in the T2DM mice. (a) Total cholesterol (TC), (b) Serum triglyceride (TG), (c) Low‐density lipoprotein cholesterol (LDL‐C), (d) High‐density lipoprotein cholesterol (HDL‐C). Values are expressed as mean ± SD (*n* = 6). *p < .05, **p < .01 compared with the control group; ^#^ *p* < .05, ^##^ *p* < .01 versus the T2DM group.

Serum biochemical indexes were measured after a 16‐week intervention. The levels of serum TC, LDL‐C, and HDL‐C showed a significantly upregulated tendency in the model group compared with the control group (*p* < .01, Figure 2), and the concentrations of TG in the T2DM model group showed a trend of upregulation but without statistical significance (Figure 2b). After treatment with metformin and quinoa, the levels of TC, TG, and LDL‐C were decreased in the metformin and quinoa groups relative to the model control group, and quinoa had a better adjustment effect than in the metformin group (Figure 2a–c). The levels of HDL‐C in the quinoa group showed a trend of downregulation significance while increasing in the metformin group relative to the T2DM group (*p* < .01, Figure 2d).

#### 3.1.3 Quinoa regulates pathological of pancreas and colon in the T2DM mice

**FIGURE 3:**
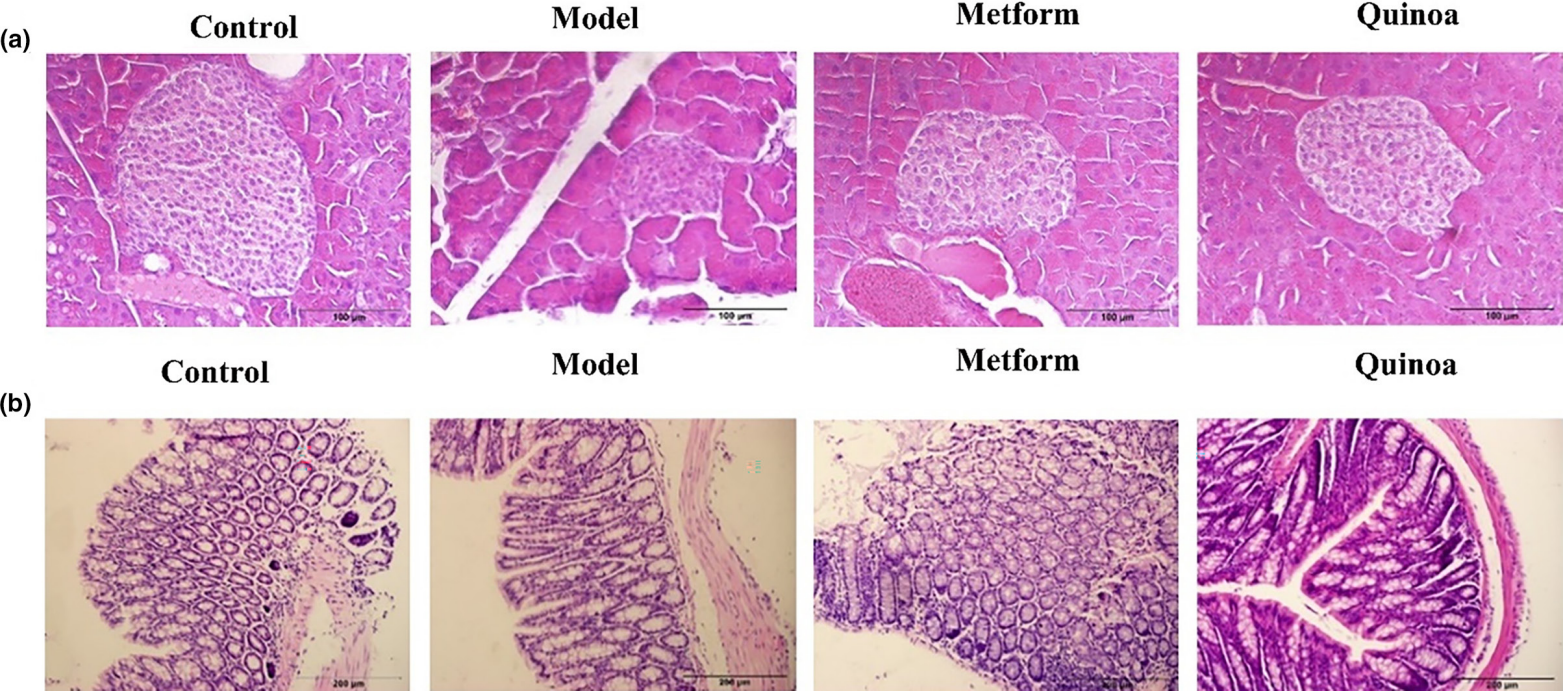
Quinoa regulates pathological of pancreas and colon in the T2DM mice. (a) HE staining of pancreas tissue, (b) HE staining of colon tissue.

The results of HE staining of pancreas and colon are shown in Figure 3, and we could observe that the islets tissue in normal control mice were large volume, regular, and round in shape. In contrast, the islet morphology was irregular and the number of islets decreased, and the tissue was severely damaged in the T2DM model group. After the intervention of metformin and quinoa, islet cell damage was significantly alleviated (Figure 3a). HE staining results of colon showed that the normal control group had intact mucosa and regular glandular arrangement, and the T2DM model group had severe deformation and mucosal abscission, whereas the metformin and quinoa groups had improved colon morphology lesions (Figure 3b). These results suggest that quinoa can protect the continuity and integrity of intestinal epithelial cells and protect the morphology of colon tissues.

### 3.2 Analysis of gut microbiota of quinoa in T2DM mice

#### 3.2.1 Quinoa could change the intestinal flora of T2DM mice

**FIGURE 4:**
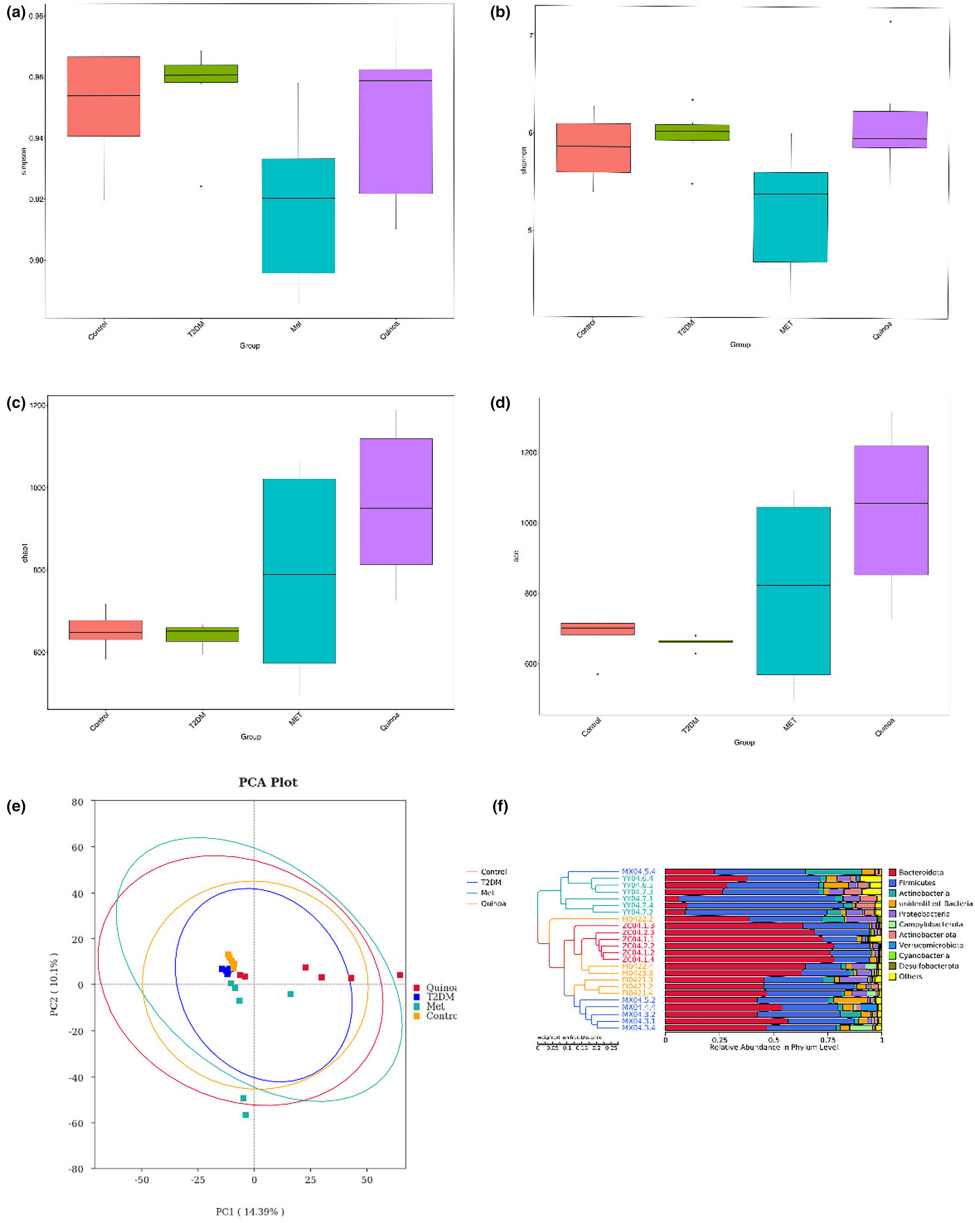
Quinoa could change the intestinal flora of T2DM mice. Alpha diversity index Simpson of quinoa in T2DM mice (a–d): (a) Simpson; (b) Shannon; (c) Chao1;(d) ACE; (e) Principal component analysis (PCA); (f) Hierarchical clustering analysis of samples.

Alpha diversity analysis could evaluate community richness and diversity in the samples. We used the Chao1 index and ACE indexes to predict community richness, the Simpson index, and Shannon index to predict the diversity of community. The results showed that the intestinal flora richness in the T2DM model group was slightly reduced compared with the control group. Relative to the T2DM group, the intestinal microflora richness of the metformin group was increased, and the intestinal microflora richness of the quinoa group was significantly increased (Figure 4a–d). Principal component analysis (PCA) was performed on the principal coordinate combination with the largest two contribution rates. The results are shown in Figure 4e. There were significant differences between the control group and T2DM, and the composition of the flora changed significantly after 16 weeks of metformin and quinoa intervention. For further prediction, further cluster analysis was performed on the samples (Figure 4f), which indicated differences in intestinal microbiome composition among groups. In conclusion, these results suggest that the intestinal flora of T2DM mice changed after quinoa intervention.

#### 3.2.2 Quinoa could adjust species abundance and structure analysis of gut microflora in T2DM mice

**FIGURE 5:**
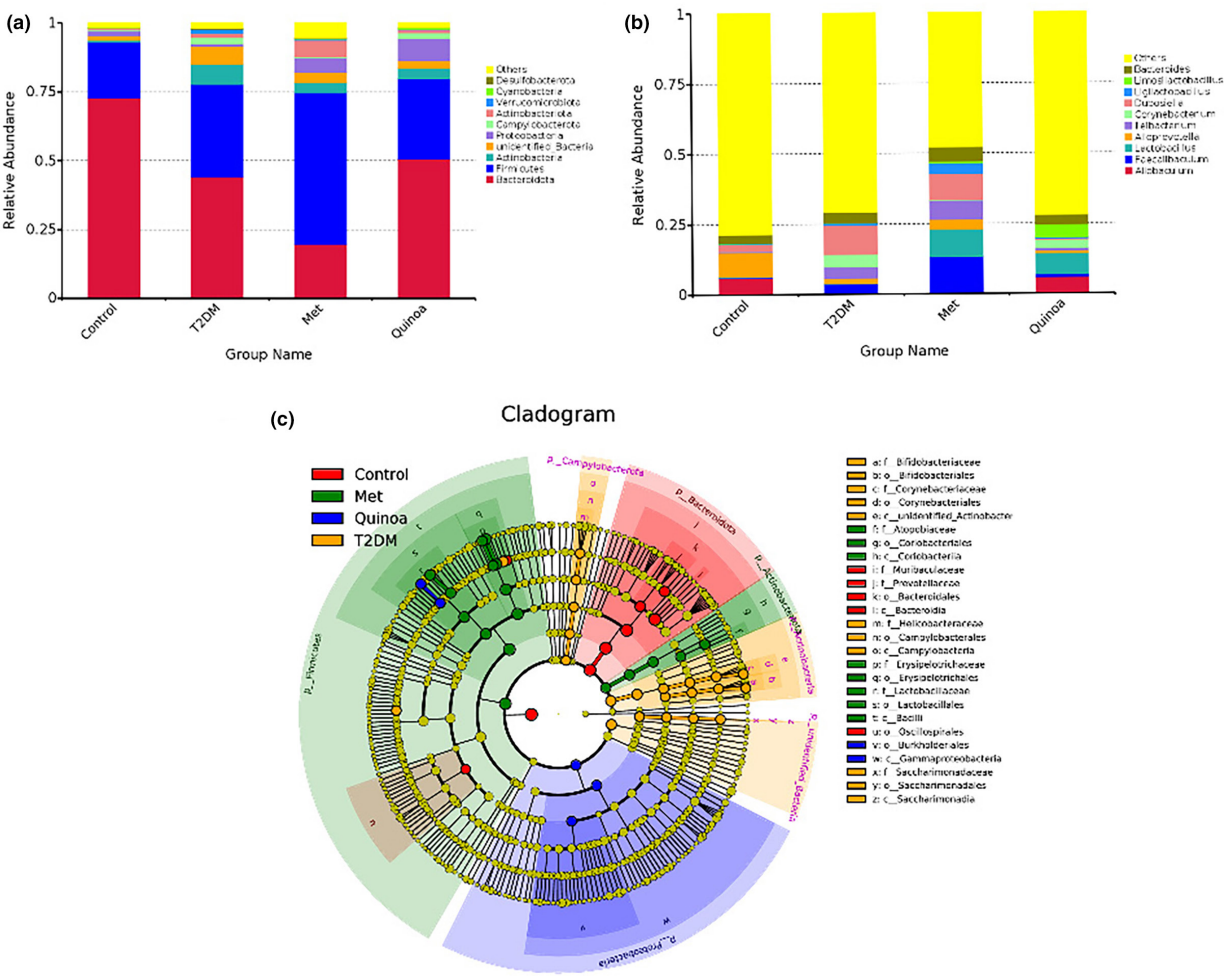
Quinoa could adjust species abundance and structure analysis of gut microflora in T2DM mice. Relative abundance of gut microbiota in four group rats: (a) At the phylum level. (b) At the class level. (c) LEfSe multilevel species hierarchical tree.

To clarify the differences in gut microbiota among the four groups further and better, we analyzed microbial species at phylum and genus levels in our study. According to the phylum level (Figure 5a), *Bacteroidota*, *Firmicutes*, *Proteobacteria*, and *Actinobacteria* were the common dominant bacteria in all groups. Compared with the normal group, the relative abundance of *Firmicutes* was increased and meanwhile the *Bacteroidetes* was decreased in the T2DM group. Relative to the model group, the proportion of *Firmicutes* in the metformin group was increased, while the *Bacteroidota* had a downward trend; the relative abundance ratio of *Bacteroides* and *Firmicutes* in the quinoa group had a tendency that is similar to the control group. At the genus level (Figure 5b), the relative abundance of *Ileibacterium*, *Corynebacterium* in the T2DM group was upregulated compared with that in the control group. Compared with the T2DM group, the proportion of *Faecalibaculum* and *Lactobacillus* in the metformin group was increased. Furthermore, the relative abundance of *Limosilactobacillus* and *Lactobacillus* was increased and the relative abundance of *Dubosiella* was reduced in the feces of quinoa mice compared with the T2DM group. LEfSe reflects the differences of enteric microorganism among the four groups directly (Figure 5c). It could be seen that the nodes in yellow area represent higher relative abundance of bacteria in the T2DM group, mainly concentrated in *Campyiobacterota* and *Actinobacteria* phylum. The nodes in blue represent the relative abundance of bacteria in the quinoa group, mainly consisting of *Proteobacteria* and *Lactobacillaceae*. The nodes in the green area represent bacteria with higher relative abundance in the metformin group, mainly concentrated in phylum of *Firmicutes* and *Actinobacteriota*. In addition, it can be seen from Figure 5c that *lactobacillus* is the specific bacteria shared by the metformin group and the quinoa group.

#### 3.2.3 Function prediction of microflora and interactive analysis of intestinal microflora in T2DM mice after quinoa intervention

**FIGURE 6:**
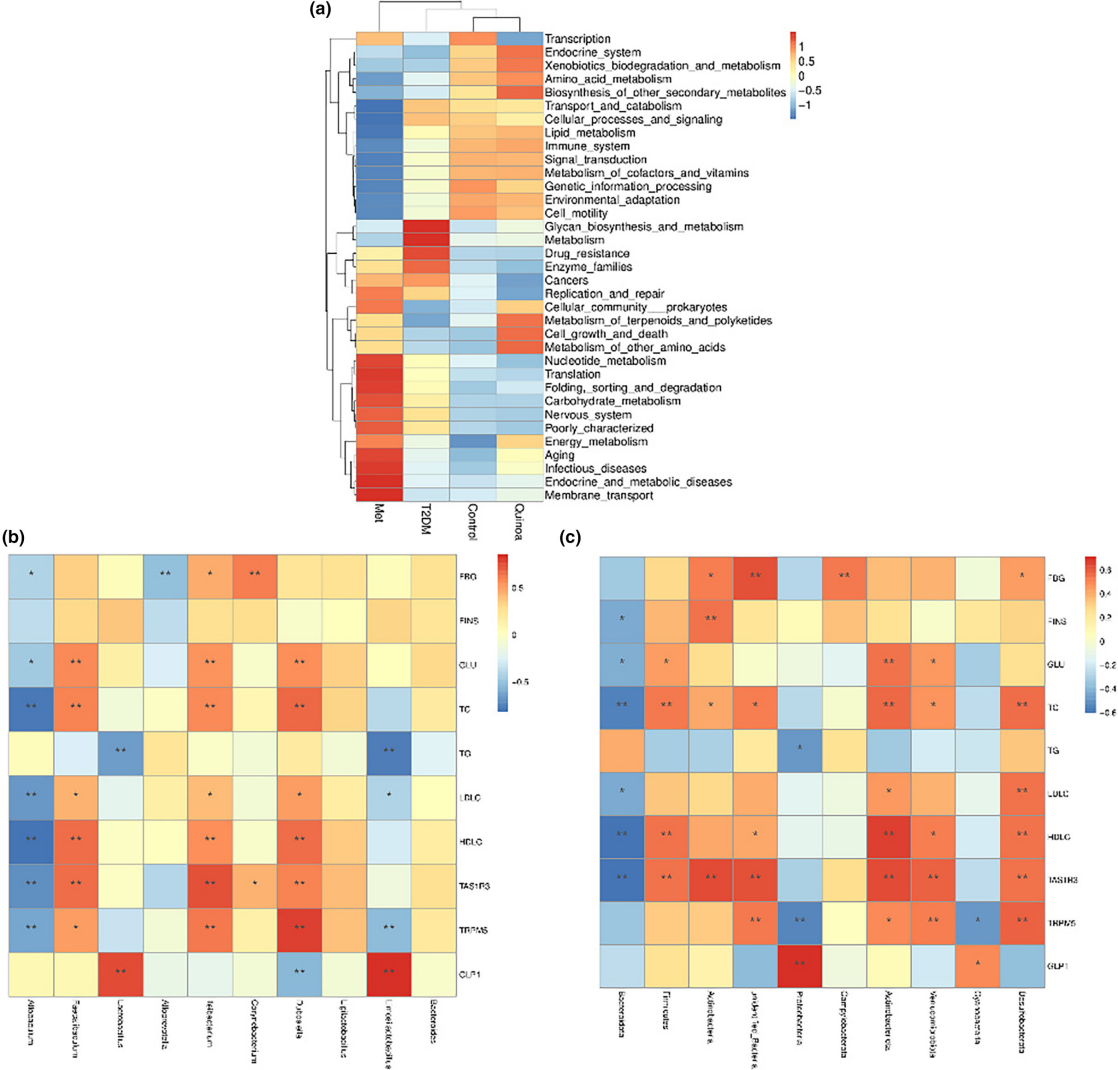
Function prediction of microflora and interactive analysis of intestinal microflora in T2DM mice after quinoa intervention. (a) Function prediction of microflora in T2DM mice after quinoa intervention. (b) Interactive analysis of intestinal microflora at the phylum level. (c) Interactive analysis of intestinal microflora at the genus level.

We can see from Figure 6a that glycan biosynthesis and metabolism, metabolism, drug resistance, enzyme families, and cancers are the higher pathways in the rats of the T2DM group compared with the control group. After the treatment of Metformin (MET) for 16 weeks, the functional prediction pathways are mainly focused on nucleotide metabolism, translation, folding, sorting and degradation, carbohydrate metabolism, nervous system, poorly characterized aging, infectious diseases, endocrine and metabolic diseases, membrane transport relative to the T2DM model group. And endocrine system, xenobiotics biodegradation and metabolism, amino acid metabolism, biosynthesis of other secondary metabolites, metabolism of terpenoids and polyketides, cell growth and death, and metabolism of other amino acids are the main pathways in the quinoa group compared with the T2DM groups.

In order to explore the effect of quinoa on type 2 diabetes mellitus, we designed an interactive analysis of intestinal microflora and biochemical indices at phylum and genus levels. As shown in Figure 6b, at the phylum level, we selected the top 10 species in relative abundance, and it could be seen through specific analysis of *Desulfobacterota* with TC, LDL‐C, HDL‐C, TAS1R3, and TRPM5; *Actinobacteriota* with GLU, TC, HDL‐C, TAS1R3, and TRPM5; besides *Bacteroidota* was negatively correlated with TC, HDL‐C, and TAS1R3 (*p* < .01); *Proteobacteria* was negatively correlated with TRPM5 and positively with GLP1 (*p* < .01). We used the same method to analyze at the genus level (Figure 6c). *Dubosiella*, *Ileibacterium*, and *Faecalibaculum* are positively correlated with GLU, TC, LDL‐C, LDL‐C, TAS1R3, and TRPM5, while *Allobaculum* was negative. In addition, TG was significantly negative and GLP‐1 was positively correlated with *Limosilactobacillus* and *Lactobacillus* (*p* < .01).

### 3.3 Results of network pharmacology of quinoa on diabetes mellitus

**FIGURE 7:**
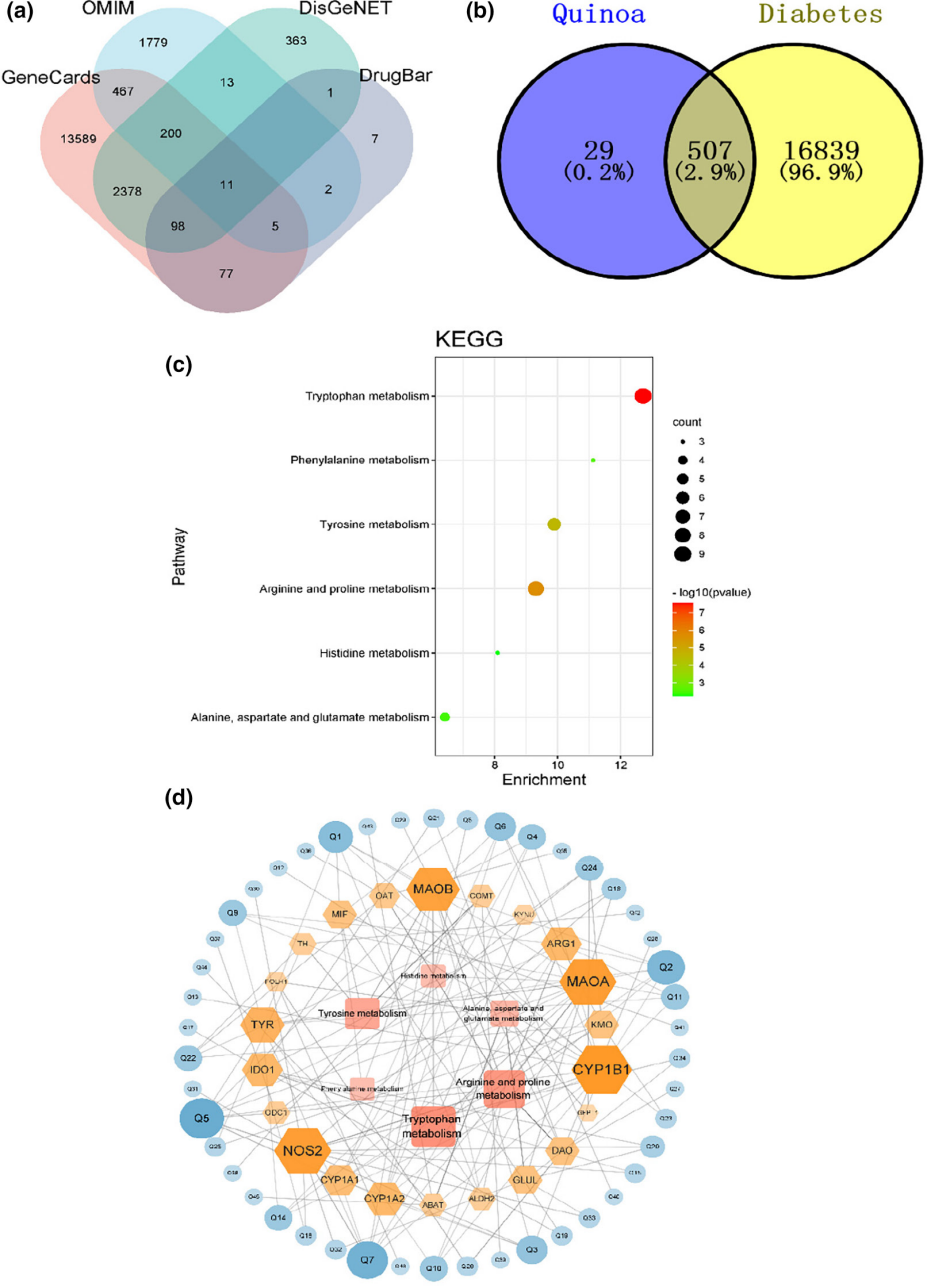
Results of network pharmacology of quinoa on diabetes mellitus. (a) Venn diagram of targets for diabetes mellitus from DrugBank, GeneCards, OMIM, and DisGeNET databases. (b) Venn diagram of targets for quinoa and diabetes mellitus. (c) Bubble map of KEGG amino acid metabolic pathway. (d) The “component‐target‐pathway” network of the six amino acid metabolic pathways.

A total of 118 chemical constituents of quinoa were collected through literature search. After intestinal absorption and drug‐like screening, 72 potential active ingredients were obtained. Based on swisstarget prediction platform, 536 potential active ingredient targets of quinoa were predicted. After retrieval from GeneCard, OMIM, DisGeNET, and DrugBank, 17, 346 diabetes‐related targets were obtained as shown in Figure 7a. Finally, we could obtain 507 for targets about quinoa in the treatment of diabetes as shown in Figure 7b. We introduced 509 targets into Metascape database for bioinformatics analysis, and obtained a total of 214 KEGG signal pathways eventually, such as neuroactive ligand–receptor interaction, pathways in cancer, and PI3K‐Akt signaling pathway (Figure S1). Among them, the pathways related to amino acid metabolism were tryptophan metabolism, arginine and proline metabolism, tyrosine metabolism, phenylalanine metabolism, histidine metabolism, alanine, aspartate and glutamate metabolism, and others as shown in Figure 7c. As shown in Figure 7d, the “component‐target‐pathway” network of the six amino acid metabolic pathways is made up of 74 nodes, 1491 sides, and 46 compounds (Table S1).

### 3.4 Results of molecular docking of quinoa on diabetes mellitus

**FIGURE 8:**
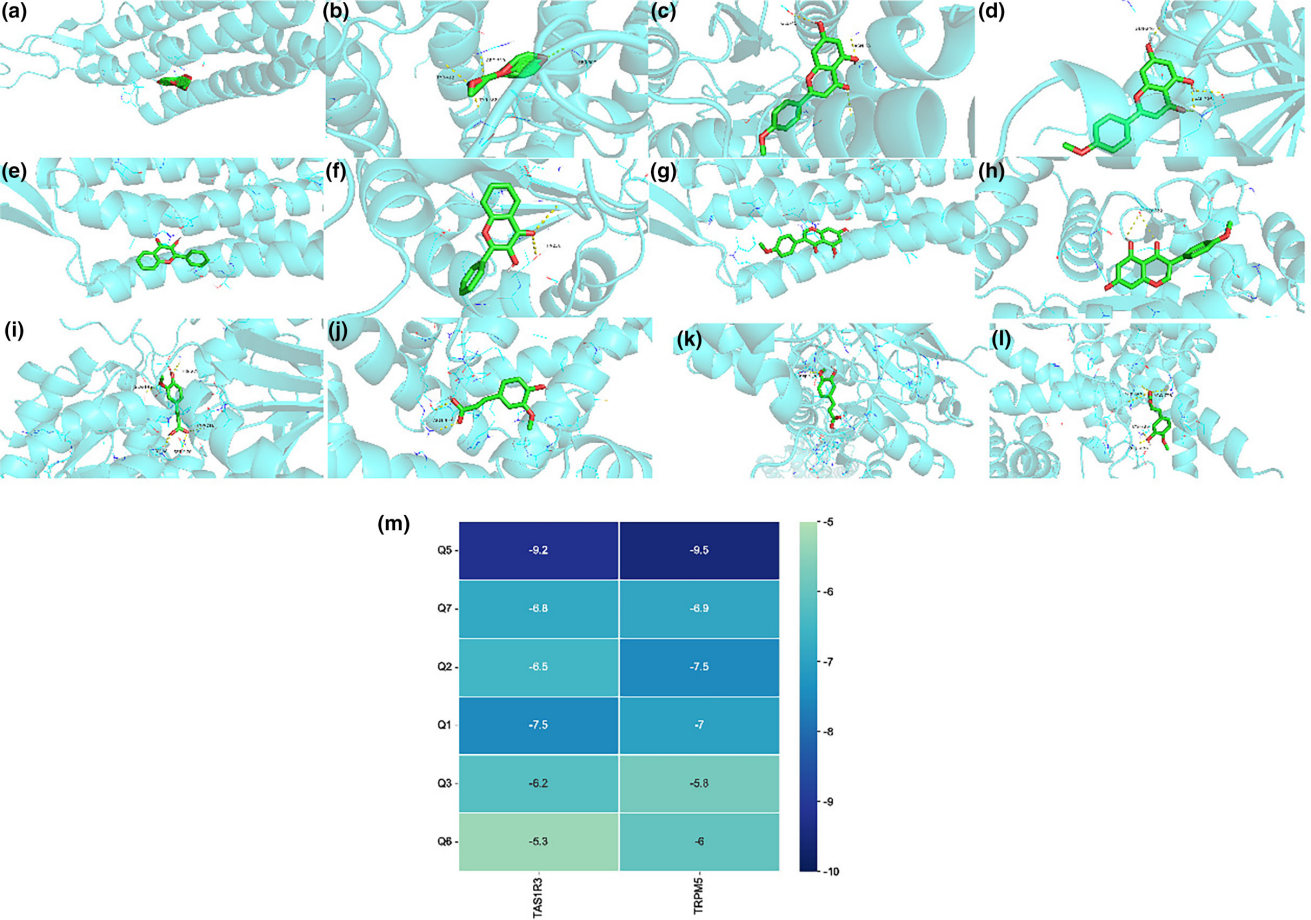
Results of molecular docking of quinoa on diabetes mellitus. (a) TAS1R3‐Apigenin‐7‐methylether, (b) TRPM5‐Apigenin‐7‐methylether, (c) TAS1R3‐Acacetin, (d) TRPM5‐Acacetin, (e) TAS1R3‐Flavonol, (f) TRPM5‐Flavonol, (g) TAS1R3‐ Biochanin A, (h) TRPM5‐ Biochanin A, (i) TAS1R3‐Ferulic acid, (j) TRPM5‐Ferulic acid, (k) TAS1R3‐Isoferulic acid, (l) TRPM5‐Isoferulic acid, (m) Heat map of molecular docking.

We chose the top six core potential compounds according to degree, closeness centrality, and betweenness centrality (Table S2) to have a docking with two taste receptors TAS1R3 and TRPM5, and 12 groups of receptor–ligand docking results were obtained (Figure 8). We could see the visual analysis from Figure 8a–l and heat map of molecular docking is shown in Figure 8m. Among them, the affinity of all combinations is less than −5 kcal/moL, and the lowest binding energy of two groups are TRPM5‐Apigenin‐7‐methylether and TAS1R3‐Apigenin‐7‐methylether, whose binding energy were −9.5 kcal/moL and −9.2 kcal/moL. The highest docking score of TAS1R3‐Linolinic acid was −5.3 kcal/moL‐1, indicating that the potential core compounds may have good binding activity to taste receptors.

### 3.5 Quinoa may improve type 2 diabetes through taste pathway

#### 3.5.1 Quinoa can regulate the expression of TAS1R3, TRPM5, and GLP‐1 in colon IHC

**FIGURE 9:**
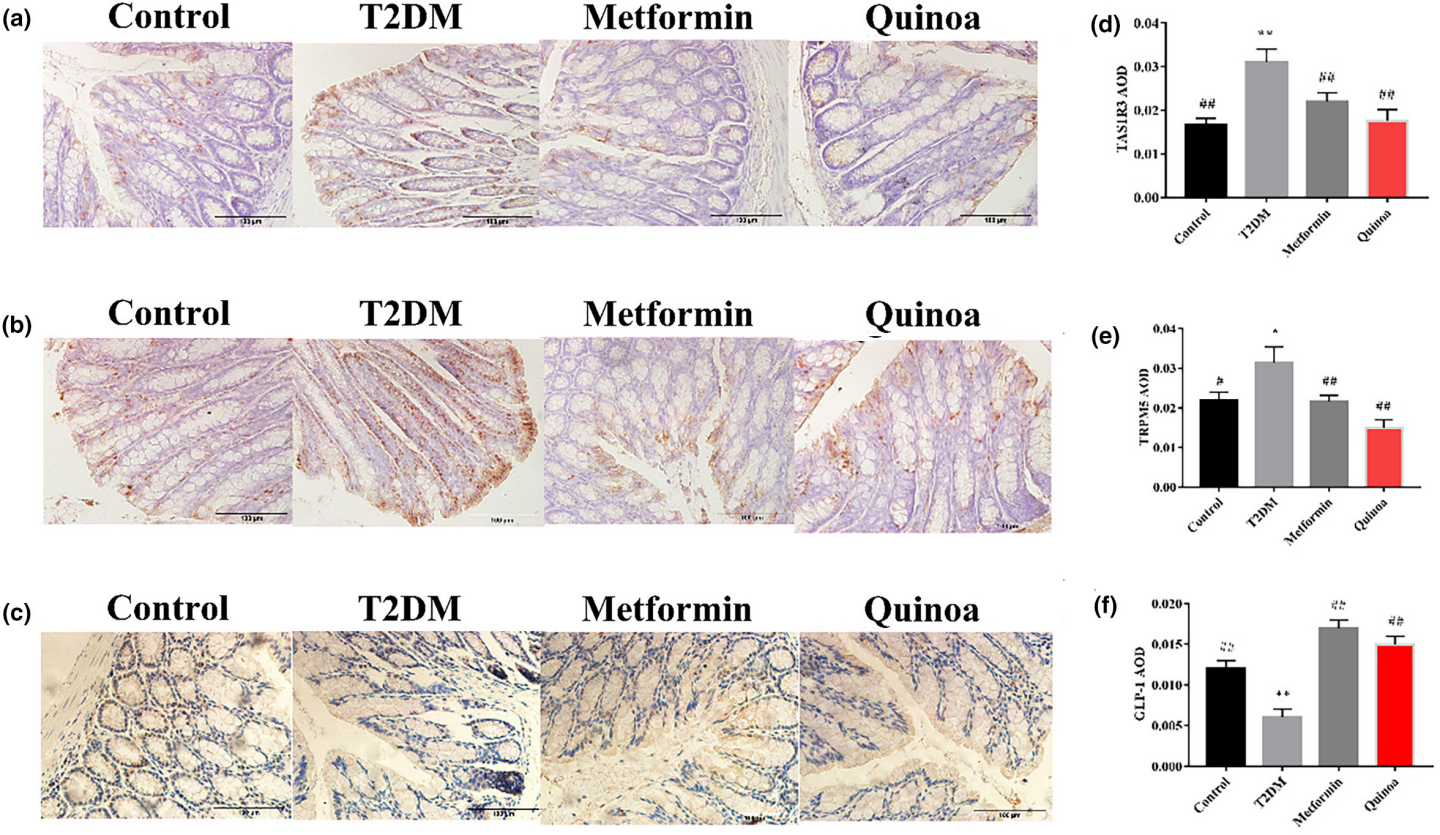
Quinoa can regulate the expression of TAS1R3, TRPM5, and GLP1 in colon immunohistochemistry (IHC). (a) IHC of TRPM5 in the colon. (b) IHC of TAS1R3 in the colon. (c) IHC of GLP‐1 in the colon. (d) AOD values of TRPM5 in the colon IHC. (e) AOD values of TAS1R3 in the colon IHC. (f) AOD values of GLP‐1 in the colon IHC. Values are expressed as mean ± SD. *p < .05, **p < .01 compared with the control group; ^#^ *p* < .05, ^##^ *p* < .01 versus the T2DM group.

We assessed TAS1R3, TRPM5, and GLP‐1 expression in colon IHC, and we could show that the expression of TAS1R3 and TRPM5 was increased in the colon of mice with T2DM (Figure 9a,b,d,e; *p* < .01), and the expression of GLP‐1 was decreased in the colon of mice with T2DM compared with that in the normal control mice (Figure 9c,f; *p* < .01). After the supplementation of metformin and quinoa for 16 weeks, the expression of TAS1R3 and TRPM5 in the colon IHC was decreased (*p* < .01), and the expression of GLP‐1 was increased (*p* < .01), and there was no statistical significance between metformin and quinoa groups.

#### 3.5.2 Quinoa can regulate the expression of TAS1R3, TRPM5, and GLP‐1 in colon Western blot

**FIGURE 10:**
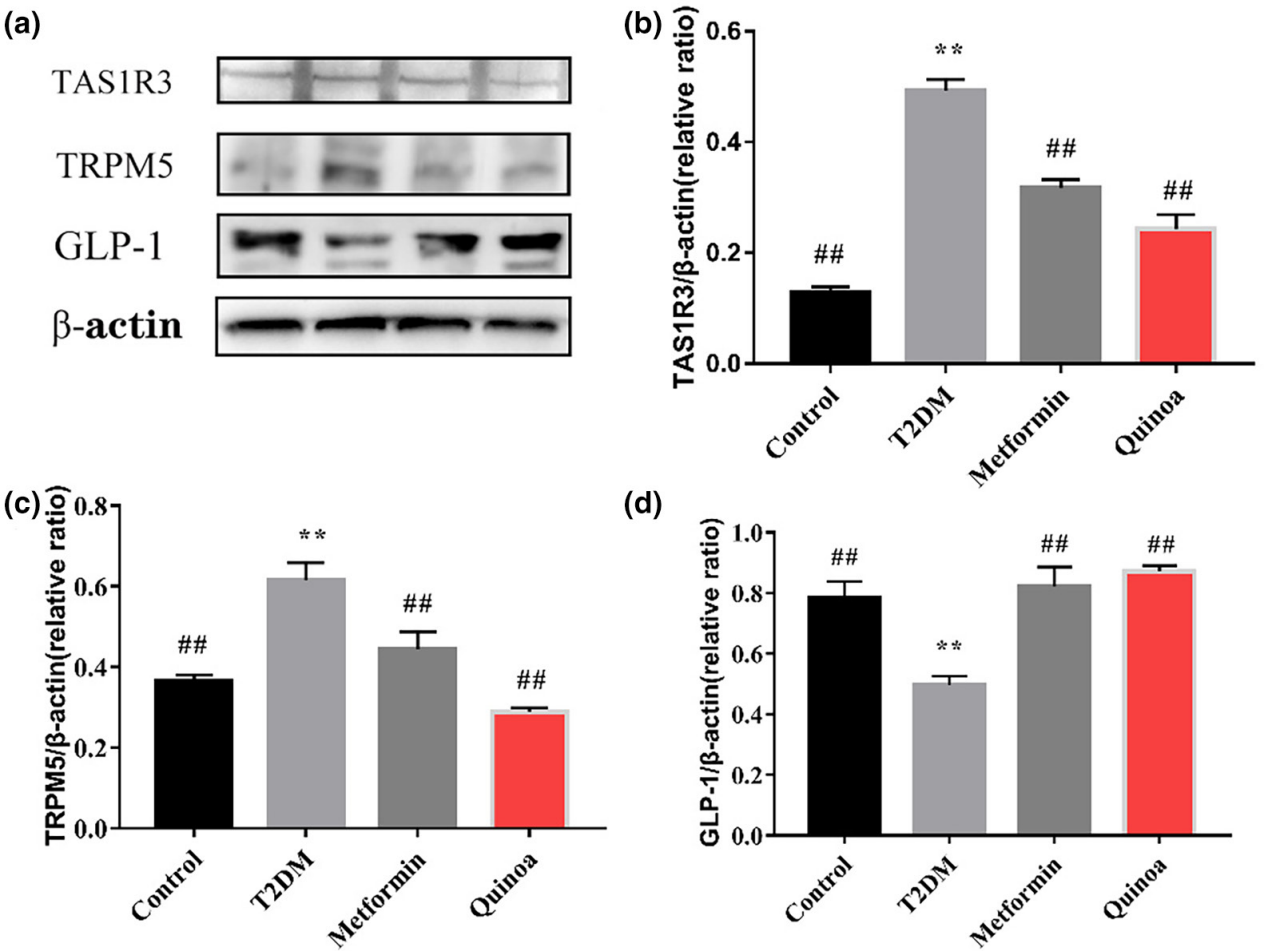
Quinoa can regulate the expression of TAS1R3, TRPM5, and GLP1 in colon Western Blot. (a) Western blot of TAS1R3, TRPM5, and GLP‐1 in the colon. (b) Quantitative assessment of the Western blot analysis results of TAS1R3 in the colon. (c) Quantitative assessment of the Western blot analysis results of TRPM5 in the colon. (d) Quantitative assessment of the Western blot analysis results of GLP‐1 in the colon. Values are expressed as mean ± SD. *p < .05, **p < .01 compared with the control group; ^#^ *p* < .05, ^##^ *p* < .01 versus the T2DM group.

It can be observed from Figure 10 that there was a significant elevation of TAS1R3 and TRPM5 in the T2DM group compared with the control group (Figure 10a–c). The expression of TAS1R3 and TRPM5 decreased significantly in the metformin group and quinoa group relative to the T2DM mice of the group (*p* < .01). Further analysis of the GLP‐1 in colon revealed that in the T2DM group, the level of GLP‐1 was lower than in the control group, whereas these changes were reversed by metformin and quinoa, and quinoa had a better regulatory effect compared with metformin (Figure 10a,d, *p* < .01).

## 4 DISCUSSION

Quinoa is considered a pseudocereal for botanical reasons which belongs to the Chenopodiaceae family (Wang, Guo, et al., 2022). As the “Golden Grain,” quinoa is rich in diverse nutrients including proteins, dietary fiber, and minerals (Angeli et al., 2020; Dakhili et al., 2019; Konishi et al., 2004; Zhu, 2020). As a kind of functional food, quinoa is an excellent example to decrease the risk of various diseases and exert health‐promoting effects (Pathan & Siddiqui, 2022). Research indicated that quinoa has a good regulatory effect on hyperglycemia which is caused by diabetes (Tang & Tsao, 2017). However, there is little evidence of the specific mechanism of its regulation of microflora and hypoglycemia in T2DM mice. Therefore, this study aimed to elucidate the regulatory effects and mechanisms of quinoa on hyperglycemia and intestinal microflora in diabetic mice which were induced by HFD and STZ.

In this study, quinoa could significantly ameliorate the symptoms of hyperglycemia and the abnormalities of related biochemical indexes in HFD/STZ‐induced diabetic mice, showing an excellent hypoglycemic effect. Abnormal glucose metabolism in diabetes is closely related to lipid metabolism. In addition, the treatment with quinoa can reduce the levels of TC, TG, and LDL‐C, as previously confirmed (An et al., 2021). Therefore, more attention should be paid to hypoglycemic foods. As studies have shown (Noratto et al., 2019), quinoa could ameliorate dyslipidemia in mice with type 2 diabetes. And quinoa has a significant effect on the improvement of blood glucose. Moreover, histopathological staining results showed that HFD/STZ‐induced islet and pancreas were damaged in type 2 diabetic mice, which could result in impaired insulin secretion, leading to the occurrence of hyperglycemia (Clark et al., 1988). After quinoa treatment, the damaged pancreas and colon tissues of mice were significantly recovered. In conclusion, our study demonstrated that quinoa has an improved effect on HFD/STZ‐induced T2DM mice.

Intestinal microbiota due to its important role is known as the second human genome (Chen, Yuan, et al., 2021) and is closely related to type 2 diabetes (Cunningham et al., 2021). Many researches have verified that *Firmicutes* and *Bacteroidetes* were vital to endocrine diseases such as obesity and diabetes (Gong et al., 2021; Wang, Tao, et al., 2022). In this study, at the phylum level, we found that the ratio of *Firmicutes*/*Bacteroidetes* increased in the model group compared to the normal group as previously said (Chen et al., 2020). After quinoa supplementation, the proportion of *Firmicutes*/*Bacteroidetes* decreased and showed a trend that resembled the control group. Thus, supplementation of quinoa could improve the chances of T2DM microorganisms significantly. At the genus level, the relative abundance of *Limosilactobacillus* and *Lactobacillus* in the quinoa group was increased compared with the model group. At the same time, *Dubosiella* showed a trend of decrease. The research showed that *Limosilactobacillus* could affect the levels of various amino acids and their derivatives in the plasma of mice (Liu, Tian, et al., 2022). *Lactobacillus* is widely regarded as a beneficial bacterium, and Ricardo et al. showed that *Lactobacillus* could further alter tryptophan levels by inhibiting indoleamine 2, 3‐dioxygenase levels (Valladares et al., 2013). Moreover, studies have shown that *Dubosiella* may be related to branched‐chain amino acid metabolism (Bao et al., 2022). We also find that *Li*mosilactobacillus and *Lactobacillus* belong to the *Lactobacillus* family, which is consistent with LEfSe results. In addition to this, LEfSe analysis indicated that the specific bacteria of the quinoa group were also concentrated in *Proteobacteria*. In the study of Del Hierro et al. 2020 (Navarro del Hierro et al., 2019), it was also shown that quinoa extract could increase the abundance of *Lactobacillus*, which was the same as in our study. There are some studies which also indicate that *Lactobacillus* can adjust blood sugar levels as beneficial bacteria (Youn et al., 2021). Lactobacillus plays an important role in improving blood sugar in many ways. For example, the increase of organic acids such as lactic acid and acetic acid leads to the decrease of intestinal pH value. Through the enrichment of lactobacillus, it further inhibits the reproduction of harmful bacteria, regulates the intestinal microecological balance, improves the intestinal mucous membrane barrier function, and ultimately effectively prevents the occurrence of diabetes (Van Gossum et al., 2007). It is also demonstrated that the abundance of Proteobacteria in the fecal microbial of treated with quinoa was also higher (Liu et al., 2018). In our study, after the intervention of quinoa, the content of Lactobacillus increased significantly compared with the diabetic model group. However, the effect of Proteobacteria after quinoa supplementation is still unclear in current studies. We look forward to further research.

Correlation analysis of microflora with blood indices and taste pathways indicated that *Actinobacteriota*, *Desulfobacterota*, *Ileibacterium*, and *Dubosiella* are positively correlated with biochemical indexes such as GLU, TC, LDL‐C, HDL‐C, and the TAS1R3/TRPM5 taste pathway, indicating that the increase of these bacteria may have a possibility of regulating the TAS1R3/TRPM5 pathway. However, it has not yet been found what explicitly links these bacteria to taste receptors. We daringly conjecture that the rise of *Actinobacteriota*, *Desulfobacterota*, *Ileibacterium*, and *Dubosiella* is closely related to the occurrence of type 2 diabetes. Studies have shown that intestinal cells can regulate taste receptors by positive feedback after sensing microbial infection, thus affecting immune response (Howitt et al., 2016). Furthermore, Glp‐1 was positively correlated with *Lactobacillus* and *Limosilactobacillus*, which confirmed the hypoglycemic effect of *Lactobacillus* further and better. In conclusion, these results indicated that quinoa regulates metabolic balance by regulating the diversity and integrity of microflora rather than a single microflora. And the prediction of microbial community function indicates that quinoa is significantly enriched in the metabolism of amino acids and other aspects.

As a kind of metabolic disease, diabetes has a complex pathogenesis. To better verify quinoa's effect on diabetes, we drew on a promising research approach called network pharmacology. It can be concluded that quinoa plays a role in regulating diabetes through multi‐target, multi‐pathway, and there are multiple pathways related to amino acid metabolism in the KEGG pathway analysis, which is consistent with the function prediction in intestinal flora. The complex relationship between amino acid metabolism and diabetes is self‐evident. It has been pointed out that taste receptors TAS1R1/TAS1R3 can mediate the signal transduction of amino acid induction in some studies (Tolhurst et al., 2012). For further validation of the interaction between the taste pathway and quinoa, we used molecular docking to verify the binding energy between the key compounds obtained from the “compound target pathway” network and related taste proteins, and the results suggest that apigenin‐8‐methylether and other compounds have a good binding activity to taste receptors, which maybe the key components of quinoa to alleviate diabetes. Related studies have also pointed out that apigenin as a kind of flavonoids could play a hypoglycemic role through antioxidation (Bai et al., 2019), which provides key scientific support for subsequent research. To sum up, our study provides strong scientific evidence to confirm that quinoa improves diabetes further and better.

The molecular biology results and molecular docking predictions are mutually consistent. Taste receptors have been shown to regulate glucose transporter (GLUTs) in intestinal (Mace et al., 2007). In the taste field, umami and sweetness are both of the basic tastes. Umami is thought to be part of the taste of amino acid, which can reflect the content of protein in the food (Iwata et al., 2014). Quinoa is rich in protein and amino acids. Taste receptors belong to G‐protein‐coupled receptors, and TAS1R3 as one of the taste receptors not only exists in the oral taste buds but is also found in the intestinal mucosa and hypothalamus (Lee & Owyang, 2017; Loper et al., 2015). Studies have shown that TAS1R2‐TAS1R3 and TAS1R1‐TAS1R3 heterodimers are used to sense organic compounds such as sugars and amino acids (Ahmad & Dalziel, 2020). Taste receptors bind to high concentrations of nutrients and activate the Ca^2+^ channel through G protein‐coupled receptors and TRPM5 for signal transduction (Zhang et al., 2007). Both belong to type II taste cells, sweet, and umami taste signal transduction has a similar signal transduction pathway. Nutrients in the gastrointestinal tract bind to taste receptors and activate phospholipase C (PLC), leading to phosphatidylinositol 4,5‐bisphosphate (PIP2) hydrolyze. PIP2 is located on the plasma membrane through gustducin, promoting Ca^2+^ release and leading to the activation of downstream molecules (TRPM5). This results in the release of GLP‐1 and gastric inhibitory polypeptide (GIP) (Le Gléau et al., 2021). However, the mechanism of nutrients such as sugars and amino acids leading to the release of gastrointestinal hormones is not clear. We look forward to further clarification. Given the importance of taste receptors in nutrient sensing and glucose homeostasis, we expected to explore the effects of quinoa on taste receptor pathways in diabetic mice. Our results show that treatment with quinoa can downregulate the related protein expression in diabetic mice. As a part of amino acids, BCAAs levels have a great influence on the venture of diabetes mellitus (Newgard, 2012; Okekunle et al., 2017). In our study, the function prediction of amino acid metabolism in the normal group and quinoa group was higher than that in the diabetic group. Cummings, Williams et al. demonstrated that adjusting the proportion of BCAAs in diet can effectively alleviate obesity and insulin resistance (Cummings et al., 2018). However, the mechanism of interaction between BCAAs metabolism and type 2 diabetes mellitus is still unclear.

Therefore, it is reasonable to assume the hypoglycemic effects of quinoa on HFD/STZ‐induced diabetic mice: (1) quinoa could modulate gut microbiota which is imbalanced by T2DM and regulates glucose metabolism ultimately. (2) Quinoa plays a hypoglycemic role through multi‐aspects. (3) Quinoa can further improve hyperglycemia by regulating amino acids metabolism through nutrient perception.

## 5 CONCLUSION

In our study, quinoa could regulate the microflora disorder in diabetic mice which was induced by HFD combined with STZ, and has the effect of alleviating hyperglycemia. The prediction of network pharmacological results showed that quinoa plays a hypoglycemic role through multi‐target, multi‐pathway, and multi‐component. Therefore, it is expected to promote human health as an auxiliary hypoglycemic food.

## AUTHOR CONTRIBUTIONS

**Chun‐Yan Zheng:** Conceptualization (equal); data curation (equal); formal analysis (equal); investigation (lead); methodology (lead); writing – original draft (lead); writing – review and editing (equal). **Tian An:** Conceptualization (lead); data curation (equal); formal analysis (equal); methodology (equal); writing – original draft (equal). **Zheng Ting Liang:** Conceptualization (equal); data curation (equal); formal analysis (lead); methodology (equal); writing – original draft (equal). **Bo‐Han Lv:** Supervision (equal); writing – review and editing (equal). **Yu Tong Liu:** Supervision (equal); writing – review and editing (equal). **Xue‐Hong Hu:** Conceptualization (equal); writing – review and editing (equal). **Yue‐Lin Zhang:** Methodology (equal); project administration (equal). **Nan Nan Liu:** Writing – review and editing (equal). **Si‐Yu Tao:** Writing – review and editing (equal). **Ru Xue Deng:** Writing – review and editing (equal). **Jia‐Xian Liu:** Resources (lead); writing – review and editing (equal). **Guangjian Jiang:** Conceptualization (equal); funding acquisition (lead); resources (equal); supervision (lead); writing – review and editing (equal).

## FUNDING INFORMATION

This work was supported by Grants from the National Natural Science Foundation of China (81774171, 82260931) and Horizontal subject of Zhong Fu Boai Health management (Beijing) Co., Ltd (2180071720024).

## CONFLICT OF INTEREST STATEMENT

The authors declare that they have no conflict of interest.

## ETHICS STATEMENT

The study protocol was approved by the Animal Care and Management Committee of the Beijing University of Chinese Medicine. All manipulations were at the request of the guidelines of the Animal Care Committee.

## Supporting information


Appendix S1

## Data Availability

All data analyzed during the current study are available from the corresponding author on reasonable request.
