# Supplementary material for: Revealing the mechanism of quinoa on type 2 diabetes based on intestinal flora and taste pathways
Source: Food Sci Nutr. 2023 Sep 23;11(12):7930–45. doi: 10.1002/fsn3.3710 (PMC10724620; doi:10.1002/fsn3.3710)
Supplement: Supplementary file 1 — Appendix S1 [file FSN3-11-7930-s001.docx]

# **Supplementary materials to ‘*Revealing the mechanism of quinoa on type 2 diabetes based on intestinal flora and taste pathways’***

# Chun-yan Zheng , Tian An, Bo-han Lv, Yu-tong Liu, Xue-hong Hu, Yue-lin Zhang，Nan-nan Liu, Si-yu Tao, Ru-xue Deng, Jia-xian Liu and Guang-jian Jiang

Table S1. Q List of names and components

| Q name | Component name |
| --- | --- |
| Q1 | Biochanin A |
| Q2 | Flavonol |
| Q3 | Ferulic acid |
| Q4 | Anthocyanin |
| Q5 | Apigenin-7-methylether |
| Q6 | Isoferulic acid |
| Q7 | Acacetin |
| Q8 | Caffeic acid |
| Q9 | Apigenin |
| Q10 | Quercetin |
| Q11 | Kaempferol |
| Q12 | Emodin |
| Q13 | Questin |
| Q14 | Isorhamnetin |
| Q15 | Gypsogenin |
| Q16 | 20-hydroxyecdysone |
| Q17 | Phloroglucinol |
| Q18 | Trans oleic acid |
| Q19 | Tryptophan |
| Q20 | Makisterone |
| Q21 | Cinnamic acid |
| Q22 | Methionine |
| Q23 | Isoleucine |
| Q24 | Histidine |
| Q25 | Tyrosine |
| Q26 | Xylic acid |
| Q27 | Benzoic acid |
| Q28 | Salicylic acid |
| Q29 | P-hydroxybenzoic acid |
| Q30 | Phytolaccagenic acid |
| Q31 | Oleic acid |
| Q32 | Linoleic Acid |
| Q33 | linolenic acid |
| Q34 | Hederagenin |
| Q35 | Leucine |
| Q36 | Valine |
| Q37 | Lysine |
| Q38 | Gallic acid |
| Q39 | 2,5-dihydroxybenzoic acid |
| Q40 | Protocatechuic acid |
| Q41 | P-coumaric acid |
| Q42 | Phenylalanine |
| Q43 | 4-Aminobutyric acid |
| Q44 | Threonine |
| Q45 | L-Alanine |
| Q46 | Vitamin B5 |

Table S2. the top 6 core potential compounds according degree， closeness centrality and betweenness centrality

| **Compound name** | **Degree** | **Closeness Centrality** | **Betweeness Centrality** |
| --- | --- | --- | --- |
| Apigenin-7-methylether | 9 | 0.354368932 | 0.095071767 |
| Acacetin | 8 | 0.341121495 | 0.062068765 |
| Flavonol | 7 | 0.296747967 | 0.01190209 |
| Biochanin A | 6 | 0.299180328 | 0.027129978 |
| Ferulic acid | 5 | 0.331818182 | 0.029396911 |
| Isoferulic acid | 5 | 0.317391304 | 0.020611241 |


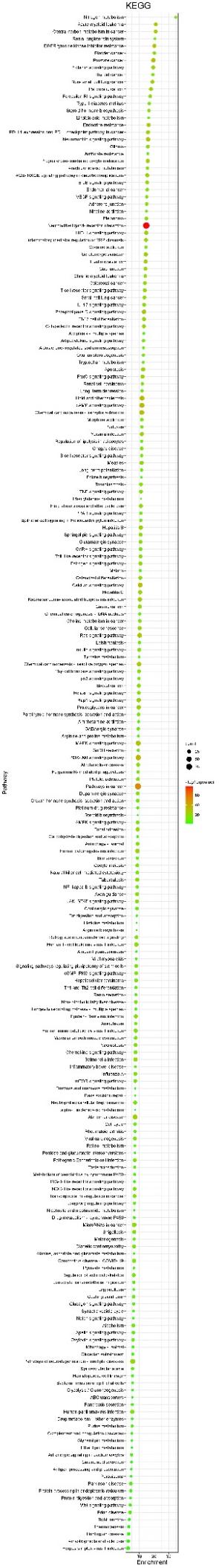


FIGURE S1. Bubble map of KEGG pathway enrichment analysis of quinoa with diabetes.
